# Lousy grouse: Comparing evolutionary patterns in Alaska galliform lice to understand host evolution and host–parasite interactions

**DOI:** 10.1002/ece3.6545

**Published:** 2020-07-18

**Authors:** Andrew D. Sweet, Robert E. Wilson, Sarah A. Sonsthagen, Kevin P. Johnson

**Affiliations:** ^1^ Department of Entomology Purdue University West Lafayette IN USA; ^2^ US Geological Survey Alaska Science Center Anchorage AK USA; ^3^ Illinois Natural History Survey Prairie Research Institute University of Illinois Champaign IL USA

**Keywords:** coevolution, genomics, *Goniodes*, grouse, *Lagopoecus*, ptarmigan

## Abstract

Understanding both sides of host–parasite relationships can provide more complete insights into host and parasite biology in natural systems. For example, phylogenetic and population genetic comparisons between a group of hosts and their closely associated parasites can reveal patterns of host dispersal, interspecies interactions, and population structure that might not be evident from host data alone. These comparisons are also useful for understanding factors that drive host–parasite coevolutionary patterns (e.g., codivergence or host switching) over different periods of time. However, few studies have compared the evolutionary histories between multiple groups of parasites from the same group of hosts at a regional geographic scale. Here, we used genomic data to compare phylogenomic and population genomic patterns of Alaska ptarmigan and grouse species (Aves: Tetraoninae) and two genera of their associated feather lice: *Lagopoecus* and *Goniodes*. We used whole‐genome sequencing to obtain hundreds of genes and thousands of single‐nucleotide polymorphisms (SNPs) for the lice and double‐digest restriction‐associated DNA sequences to obtain SNPs from Alaska populations of two species of ptarmigan. We found that both genera of lice have some codivergence with their galliform hosts, but these relationships are primarily characterized by host switching and phylogenetic incongruence. Population structure was also uncorrelated between the hosts and lice. These patterns suggest that grouse, and ptarmigan in particular, share habitats and have likely had historical and ongoing dispersal within Alaska. However, the two genera of lice also have sufficient dissimilarities in the relationships with their hosts to suggest there are other factors, such as differences in louse dispersal ability, that shape the evolutionary patterns with their hosts.

## Graphical abstract

Genomic data from Alaska grouse species and their associated feather lice suggest a mixed history of host switching and codivergence between these organisms. The results give insight into the ecological and evolutionary history of grouse in Alaska, and also help us understand how hosts and their parasites interact over time.

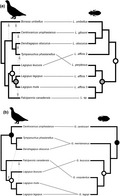

## 1 INTRODUCTION

Many parasite taxa are tightly linked with one or a few host species. These organisms spend their entire lives on a host and depend on their host for nutrients, survival, and reproduction (Poulin, 2011; Poulin, Krasnov, & Mouillot, 2011; Rohde, 1979). Because of these close associations, permanent parasites are often predicted to have evolutionary patterns of diversification that are congruent with the patterns of their hosts. This logic formed the basis for several “rules of parasitism” developed in the early 20th century, such as Fahrenholz's Rule of mirroring host–parasite phylogenies (Eichler, 1948; Fahrenholz, 1913). Similar work even proposed that parasite phylogenies could be used as a proxy for their hosts' phylogeny (Harrison, 1914; Hopkins, 1942). Although subsequent research has shown that Fahrenholz's Rule and related predictions are infrequently demonstrated in natural systems (Braga, Razzolini, & Boeger, 2014; Doña et al., 2017; Fecchio et al., 2018; Hoberg & Brooks, 2008; Johnson, Williams, Drown, Adams, & Clayton, 2002; de Vienne et al., 2013), the concept of using host–parasite comparisons to understand the evolutionary and ecological history of the hosts and, more generally, the evolutionary history of host–parasite interactions remains a useful approach.

Host–parasite comparisons can be informative at both macro‐ and microevolutionary scales. At the macroevolutionary scale, comparing host and parasite phylogenies can indicate factors that promote host switching or codivergence. These factors often highlight behavioral, morphological, or ecological traits of the hosts. For example, phylogenetic comparisons between bats and their mites suggest roosting behavior in the bats has promoted rampant host switching over evolutionary time (Bruyndonckx, Dubey, Ruedi, & Christe, 2009). Similarly, frequent host switching of the monogenean parasites of teleost fish is perhaps predicted by the social behavior of the hosts (Desdevises, Morand, Jousson, & Legendre, 2002). In a different scenario, the isolated habits of pocket gophers may largely account for the codiversification with their chewing lice (Hafner et al., 1994; Light & Hafner, 2007). Parasites can also provide insight into the biogeographic history of their hosts. This has been shown for a diverse set of host–parasite systems, including toucan feather lice (Weckstein, 2004), fungal parasites from southern beech trees (Peterson, Pfister, & Bell, 2010), and nematodes from mice (Nieberding et al., 2008).

Comparable to questions of biogeography over deeper evolutionary time, sampling a single parasite species or parasites from a single host species can give insights into the phylogeographic patterns of a host in a particular region (Criscione, Poulin, & Blouin, 2005; Dybdahl & Lively, 1996; Engelbrecht, Matthee, du Toit, & Matthee, 2016; du Toit, Van Vuuren, Matthee, & Matthee, 2013; Whipps & Kent, 2006). Because parasites often have faster rates of molecular evolution compared to their hosts, population genetic patterns of parasites can reveal histories of host dispersal not yet discernible in the host's genome (Hafner et al., 1994; Johnson et al., 2014; Ricklefs & Outlaw, 2010). Several types of parasites are known to demonstrate this phenomenon, including mites from bats (Speer et al., 2019), helminths from pikas (Galbreath & Hoberg, 2012), and feather lice from Galápagos hawks (Whiteman, Kimball, & Parker, 2007).

Of course, factors related to the parasites themselves also shape observed host–parasite evolutionary patterns (Sweet et al., 2018). To discern the effects of these factors, an ideal system would focus on multiple types of similar parasites associated with the same group of hosts. For example, phylogenetic comparisons between *Ficus* figs and their two different types of fig wasps indicate that wasp interactions (mutualist versus parasite) shape the evolutionary patterns in this system (Weiblen & Bush, 2002). In another case, evolutionary patterns between doves and their wing and body feather lice seem to be largely driven by difference in dispersal ability between the two types of lice (Clayton & Johnson, 2003; DiBlasi et al., 2018; Sweet & Johnson, 2018).

In this study, we use feather lice from North American grouse to understand the evolutionary histories between these different organisms, with a particular focus on lice from ptarmigan. Grouse are a monophyletic group of Galliformes (Aves: Phasianidae: Tetraoninae) (Lucchini, Hö, Klaus, Swenson, & Randi, 2001; Persons, Hosner, Meiklejohn, Braun, & Kimball, 2016) which are broadly distributed in temperate, subarctic, and Arctic regions in both North America and Eurasia, and have adapted to live in cold and high‐elevation habitats (de Juana, 1994; Short, 1967). Grouse are principally resident species with movements typically classified as localized seasonal movements (within a few kilometers of breeding areas; Brander, 1967; Fedy, Martin, Ritland, & Young, 2008; Hoffman & Braun, 1975; Hörnell‐Willebrand, Willebrand, & Smith, 2014; Johnsgard, 1983; Rodewald, 2020), but some populations do undertake short‐distance migrations (e.g., 160 km movements in northern Alaska; Irving, West, Peyton, & Paneak, 1967; Leonard, Reese, & Connelly, 2000). In addition, there does appear to be repeated instances of long‐distance dispersal events throughout the evolutionary history of Galliformes, including multiple dispersals from the New World to the Old World (Lucchini et al., 2001).

**Figure 1:**
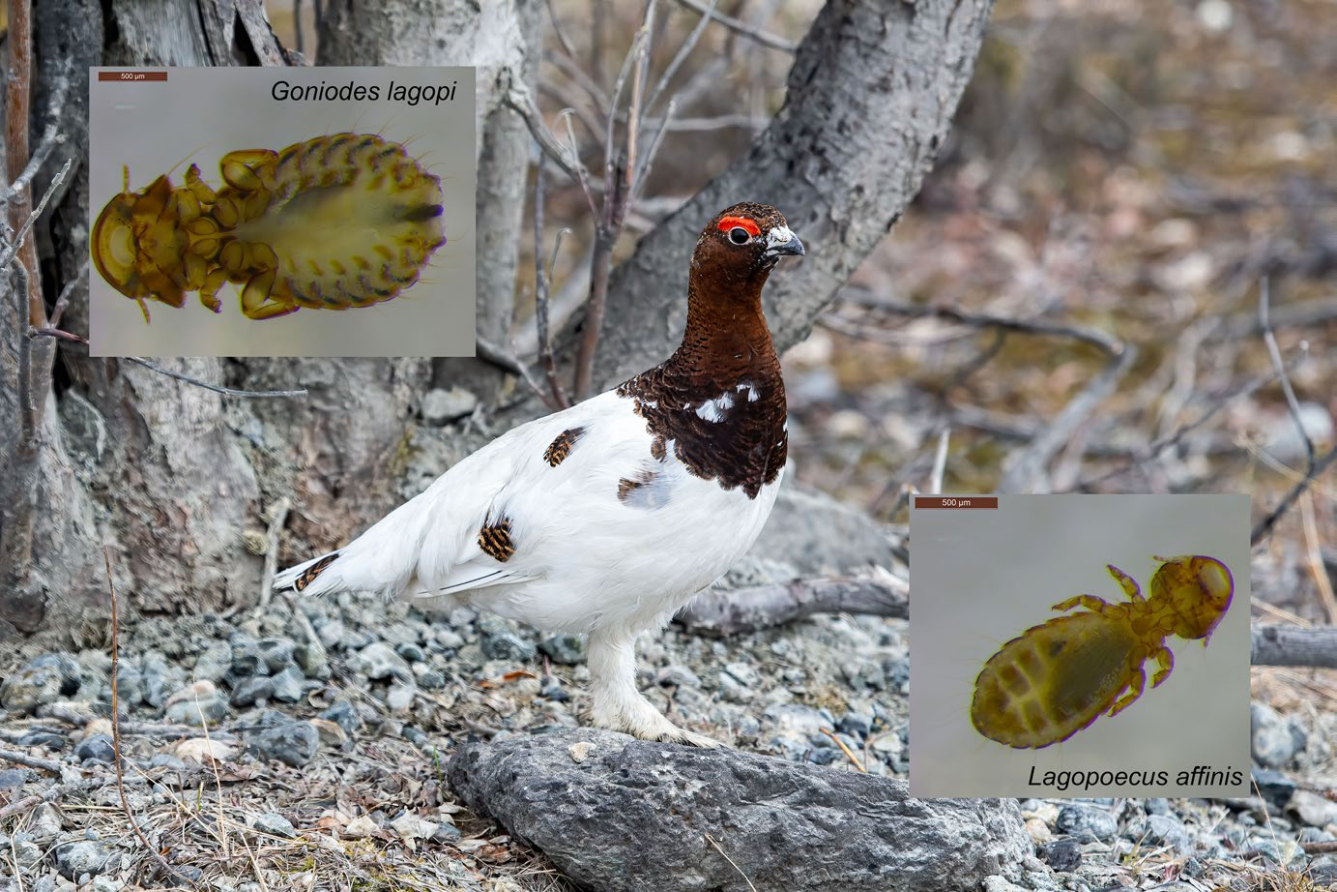
Photographs of a willow ptarmigan (*Lagopus lagopus*) and their associated lice *Goniodes* and *Lagopoecus*. Photograph credit: Sarah Sonsthagen, US Geological Survey

Ptarmigan host two different genera of lice (*Goniodes* and *Lagopoecus*) that occur with high prevalence (Figure 1). As with all feather lice (Phthiraptera: Ischnocera), both genera of ptarmigan lice are permanent and obligate parasites that eat the downy feathers on their host's body (Marshall, 1981). Lice in the genus *Goniodes* are widespread across the Galliformes (including on chickens and turkeys), whereas lice in the genus *Lagopoecus* are more restricted in their host distribution, being primarily associated with ptarmigan and other related grouse species (Price, Hellenthal, Palma, Johnson, & Clayton, 2003). Currently, there are two species of *Goniodes* (*G. lagopi* and *G. leucurus*) and a single species of *Lagopoecus* (*L. affinis*) known to be associated with ptarmigan (Price et al., 2003). When a group of birds host multiple types of feather lice, the different lice are often specialized to live in particular microhabitats on the bird's body (Johnson, Shreve, & Smith, 2012). *Goniodes* is a body louse, escaping from host preening by burrowing in the downy portions of the host's body feathers. *Lagopoecus* has the shape of a more generalized ecomorph, which can be found on feathers throughout the body and on the wings.

Here, we sequence the genomes of 51 *Goniodes* and *Lagopoecus* lice from all three ptarmigan species and five North American grouse species. From these data, we assemble hundreds of nuclear genes for phylogenetic analyses and call genome‐wide single‐nucleotide polymorphisms (SNPs) for addressing population‐level questions. For population comparisons, we also sequence SNPs of ptarmigans from multiple populations in Alaska, USA. Based on these analyses, we address two questions at different evolutionary scales. First, is there a history of host switching of lice between different species of ptarmigans and grouse, or do the birds and their lice tend to codiverge with one another? Either pattern would provide insight into the biogeography and interspecific interactions of the hosts over evolutionary time. Second, is the population genetic structure of ptarmigan in Alaska congruent with the structure of their lice? Discordance would suggest there is host movement among different host populations or that there is some other independent dispersal mechanism of the lice. Addressing these questions will clarify the coevolutionary history between Arctic Galliformes and their lice and will further highlight the utility of using parasites to understand the evolution and ecology of their hosts.

## 2 MATERIALS AND METHODS

### 2.1 Louse collection and sequencing

**Figure 2:**
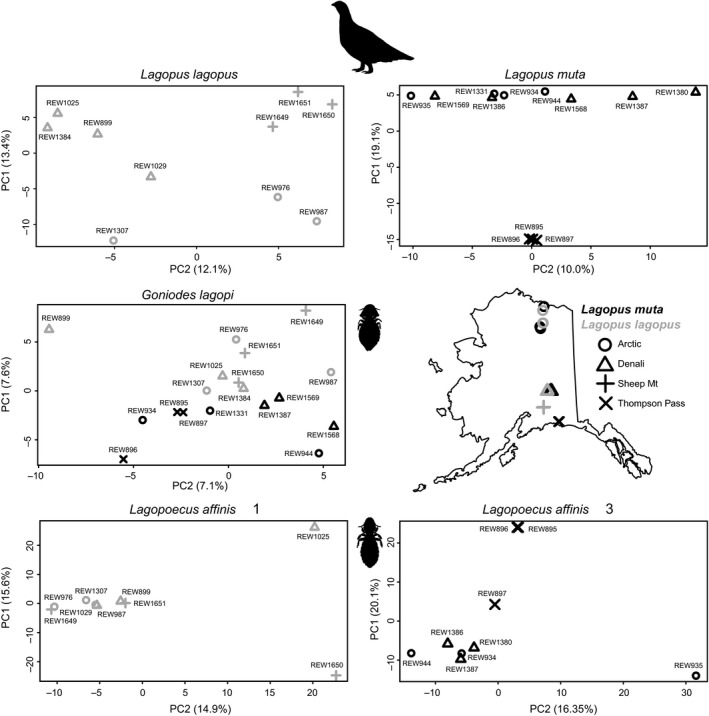
Principal component analysis (PCA) plots based on genome‐wide SNPs of two species of ptarmigans (*Lagopus muta* and *L. lagopus*) and three of their associated louse taxa (*Goniodes* and *Lagopoecus*). Sampling locations and ptarmigan populations are indicated on the map of Alaska, with shapes and shading (black or gray) corresponding to points on the PCA plots. Points on the PCA plots are also labeled according to the host codes listed in Table S1

We collected lice from ptarmigan and grouse species using the fumigation method described in Clayton and Drown (2001) or by visual inspection of the feathers. Most of these ptarmigan and grouse lice were collected from 11 localities throughout Alaska, USA, spanning ~10 latitudinal degrees. This included population‐level sampling for *Lagopoecus* and *Goniodes* lice from willow (*Lagopus lagopus*) and rock ptarmigan (*Lagopus muta*), representing three populations of rock ptarmigan (Arctic, Denali Highway (Denali hereafter), Thompson Pass) and three populations of willow ptarmigan (Arctic, Denali, Sheep Mountain) (Figure 2, Table S1). Additional samples were collected from Utah, USA. Collected lice were immediately placed in >95% ethanol and stored at −80°C as soon as possible. The lice were identified using keys and host records in Price et al. (2003).

We sequenced the genomes of 51 louse specimens, including 26 *Lagopoecus* and 25 *Goniodes* (Table 1). Of the samples for each genus, 18 (36 total) of these were lice collected from two ptarmigan species: willow (9 samples each of *Lagopoecus* and *Goniodes*) and rock ptarmigan (9 samples each of *Lagopoecus* and *Goniodes*). The remaining samples included lice from related North American grouse taxa collected in Alaska and Utah, including white‐tailed ptarmigan (*Lagopus leucura*), spruce grouse (*Falcipennis canadensis*), sharp‐tailed grouse (*Tympanuchus phasianellus*), dusky grouse (*Dendragapus obscurus*), greater sage‐grouse (*Centrocercus urophasianus*), and ruffed grouse (*Bonasa umbellus*; for *Lagopoecus* only) (Table S1). For outgroups, we included *Goniodes ortygis* (bobwhite louse), *Goniocotes chrysocephalus* (grouse louse), *Passonomedea* sp. (wood‐quail louse), *Auricotes* sp. (imperial‐pigeon louse), and *Campanulotes compar* (pigeon louse) for *Goniodes*, and *Degeeriella rufa* (falcon louse), *Cuculicola atopus* (cuckoo louse), *Colinicola docophoroides* (quail louse), and *Lipeurus caponis* (chicken louse) for *Lagopoecus*. Because each specimen was destroyed for DNA extraction, the louse specimens were photographed as vouchers (photos available from Dryad). We then extracted DNA from individual louse specimens using a Qiagen QIAamp DNA Micro kit (Valencia, CA, USA) following the modified protocol outlined in Sweet et al., (2018). Genomic DNA was quantified using a Quant‐iT dsDNA High Sensitivity assay kit (Invitrogen). Before genomic sequencing, we amplified and sequenced the mitochondrial COI gene for each sample to verify our extractions using the primer pair L6625 and H7005 (Hafner et al., 1994). PCR amplifications, cycle sequencing, and postsequencing protocols followed Sonsthagen, Talbot, and McCracken (2007). Sequences are accessioned in GenBank (MT517178–MT517227).

**Table 1:** Number of *Lagopoecus* and *Goniodes* lice sampled from different grouse species for whole‐genome sequencing

Host species	*Lagopoecus*	*Goniodes*	"Arctic"	"Denali"	"Sheep Mountain"	"Thompson Pass"
*Lagopus lagopus*	9	9	3/3	3/3	3/3	–
*Lagopus muta*	9	9	3/3	3/3	–	3/3
*Lagopus leucura*	3	3	–	–	–	–
*Centrocercus urophasianus*	1	1	–	–	–	–
*Dendragapus obscurus*	1	1	–	–	–	–
*Tympanuchus phasianellus*	1	1	–	–	–	–
*Falcipennis canadensis*	1	1	–	–	–	–
*Bonasa umbellus*	1	0	–	–	–	–

Note

For rock (*Lagopus muta*) and willow (*Lagopus lagopus*) ptarmigan, the number of lice (*Lagopoecus/Goniodes*) sampled from four populations in Alaska is also listed.

Genomic libraries were prepared with a Nextera DNA Flex kit (Illumina) or Hyper Library construction kits (KAPA Biosystems) following manufacturers' protocols and DNA input concentrations (Table S1). Libraries were quantified using quantitative PCR and Illumina library quantitation kits (KAPA Biosystems, Wilmington, MA, USA). Paired‐end, 150 bp‐read sequencing was completed on either an Illumina HiSeq4000 or NovaSeq 6000 (S4 reagent kit) (Table S1). Resulting sequences were demultiplexed with bcl2fastq v.2.20 (Illumina). We deposited all raw paired‐end reads on the NCBI SRA database (BioProject PRJNA635170; SAMN15029953–SAMN15029999). We trimmed adapters and low‐quality (phred < 28) sequences from libraries using the FASTX‐Toolkit (http://hannonlab.cshl.edu/fastx_toolkit/) and confirmed the quality of the trimmed libraries using FastQC v.0.11.5 (Babraham Bioinformatics).

### 2.2 Gene assembly and read mapping of lice

Assembling and mapping loci for phylogenomic analysis followed the workflow described in Sweet et al., (2018). Information for these assemblies (number of reads, genes assembled, depth) is available in Table S1. First, we used aTRAM v.2.1.1 (Allen, LaFrance, Folk, Johnson, & Guralnick, 2018) to assemble nuclear orthologs for one representative each of *Goniodes* and *Lagopoecus* lice (both from *Lagopus leucura* REW1396). These two lice were sequenced at higher coverage than most of the other louse samples. We targeted 1,107 single‐copy orthologous genes from the human body louse (*Pediculus humanus humanus*; Kirkness et al., 2010) using three aTRAM iterations and ABySS (Simpson et al., 2009) for de novo assembly. We then ran a postprocessing script from Allen et al. (2017) that uses Exonerate v.2.2.0 (Slater & Birney, 2005) to identify and stitch together exon regions for each assembled gene.

We assembled nuclear genes for the remaining *Goniodes* and *Lagopoecus* libraries by read mapping to the aTRAM‐assembled exons with Bowtie2 (Langmead & Salzberg, 2012). We mapped each library to sequences from the same genus, then used SAMtools and BCFtools (Li et al., 2009) to create pileup files and filter according to quality (phred ≥ 28) and depth (>10 and <100).

Finally, we used aTRAM to assemble COI for samples that did not successfully amplify using PCR. For these assemblies, we used 10% of the reads and COI from the pigeon louse *Campanulotes bidentatus* mitochondrial genome (Covacin, Shao, Cameron, & Barker, 2006) as a target.

### 2.3 Louse phylogenetic analyses

We conducted separate phylogenetic analyses for the *Goniodes* and *Lagopoecus* datasets. We aligned each gene by amino acids in PASTA (Mirarab et al., 2015) and back‐translated the aligned genes to nucleotides using a custom Python script. We removed gene alignments that did not include at least half of the taxa and used trimAL v.1.4 (Capella‐Gutiérrez, Silla‐Martínez, & Gabaldón, 2009) to mask sites containing >75% gaps. We also removed genes that contained a stop codon in the translated alignments. We aligned the COI sequences using the default settings of MAFFT (Katoh, Misawa, Kuma, & Miyata, 2002) in Geneious v.11.1.5 (Biomatters, Ltd.).

We used both coalescent and concatenated‐based approaches for phylogenetic estimation. For the coalescent analyses, we estimated maximum likelihood (ML) gene trees with RAxML v.8.2.11 (Stamatakis, 2014) using GTR + Γ substitution models. We summarized the best gene trees with ASTRAL v.4.10.5 (Mirarab & Warnow, 2015) using the local posterior probability branch support (Sayyari & Mirarab, 2016). For the concatenated analyses, we estimated the optimal gene partitioning scheme using the rcluster search in PartitionFinder v.2.1.1 (Lanfear, Calcott, Kainer, Mayer, & Stamatakis, 2014; Lanfear, Frandsen, Wright, Senfeld, & Calcott, 2016). We then ran a partitioned ML analysis in RAxML with GTR + Γ models and 250 bootstrap replicates. Finally, we used RAxML to estimate an ML phylogeny from the COI alignment using a GTR + Γ model and 500 bootstrap replicates.

Most of the louse specimens were not identified to species, so we used multiple approaches for assessing operational taxonomic units (OTUs) that potentially represent cryptic species. First, we used the COI sequence alignments as inputs into the Automatic Barcode Gap Discovery (ABGD) method (Puillandre, Lambert, Brouillet, & Achaz, 2012), which assesses OTUs by identifying gaps in the distribution of genetic distances. We ran ABGD with Jukes–Cantor (JC69), Kimura (K80), and raw distance models, each with default parameters. Second, we used the best RAxML trees from the partitioned concatenated alignments as inputs into the web version of bPTP (Zhang, Kapli, Pavlidis, & Stamatakis, 2013). We removed out‐group taxa and ran the MCMC for 100,000 generations, with thinning set to 100 and a 10% burn‐in.

### 2.4 Cophylogenetic comparisons

We used both distance‐based and event‐based approaches to test for cophylogenetic patterns between grouse and their lice. The two approaches test fundamentally different questions: distance‐based methods test whether host and parasite phylogenies are statistically congruent, whereas event‐based methods reconcile two phylogenies with specific evolutionary events, such as codivergence and host switching. In all tests, we trimmed the ML louse phylogenies to include one representative per OTU, as determined by the OTU analyses. For a host phylogeny, we used the ML tree reported in Persons et al. (2016).

We used Parafit (Legendre, Desdevises, & Bazin, 2002) and PACo (Balbuena, Míguez‐Lozano, & Blasco‐Costa, 2013) for distance‐based approaches. We converted the phylogenies to patristic distance matrices and ran both methods with 9,999 permutations and the Cailliez correction for negative eigenvalues. We ran Parafit in the R package *ape* v.5.3 (Paradis, Claude, & Strimmer, 2004), testing for the contribution of individual associations to the overall congruence while correcting for multiple tests with the Benjamini–Hochberg correction (Benjamini & Hochberg, 1995). We ran PACo in the R package *paco* v.0.3.2 (Hutchinson, Cagua, Balbuena, Stouffer, & Poisot, 2017) with the r0 method (i.e., assuming the parasite phylogeny tracks the host phylogeny) and testing for the contribution of individual associations.

We used Jane v.4.01 (Conow, Fielder, Ovadia, & Libeskind‐Hadas, 2010) as an event‐based method. This software reconciles two phylogenies by using a genetic algorithm to minimize the sum of values based on a priori costs assigned to different coevolutionary events. We ran Jane with default event costs (cospeciation = 0, duplication = 1, host switch = 2, loss = 1, fail to diverge = 1) and genetic algorithm parameters set to the following: population size = 500 and generations = 100. We also included time constraints on the host phylogeny, based on the divergence time estimates from Persons et al. (2016), to inform our cophylogenetic reconciliations. We created two time zones and forced the split between *Centrocercus* and *Dendragapus* + *Tympanuchus* (zone 1) to occur before the crown split in ptarmigans (*Lagopus*) (zone 2). The splits in the louse phylogenies were allowed to occur in either time zone, since we do not have divergence time estimates for those taxa. We then randomized the host–parasite associations 999 times to test whether the observed total cost was significantly lower than with random associations. In order to test our results against an unconstrained reconciliation, we also ran Jane without using time constraints on the host phylogeny, using the same cost and genetic algorithm parameters as in the constrained analyses.

### 2.5 Obtaining SNPs for ptarmigan and their lice

We used genome‐wide SNPs to assess population genetic structure in two species of ptarmigan (willow and rock ptarmigan) and their lice. For the lice, we called bi‐allelic SNPs for one OTU of *Goniodes lagpoi* (18 louse individuals) and two OTUs of *Lagopoecus affinis* (18 total louse individuals, 9 from each OTU) identified in the OTU analyses. These lice were sampled from three distinct regions: a northern region (“Arctic”), an interior region (“Denali”), and a south‐central region (“Sheep Mountain” for willow ptarmigan, “Thompson Pass” for rock ptarmigan). We jointly called SNPs for each OTU in SAMtools and BCFtools, with the same references used in the read‐mapping pipeline. We then used BCFtools to filter the called SNPs based on coverage, removing SNPs with depth < 10 and >100. This pipeline generated 13,676 SNPs (0.29% missing) for *G. lagopi* lice from willow and rock ptarmigan, 22,259 SNPs (0.38% missing) for *L. affinis* lice from willow ptarmigan, and 22,218 SNPs (0.48% missing) for *L. affinis* lice from rock ptarmigan.

We used double‐digest restriction‐site‐associated DNA (ddRAD) sequences to obtain SNPs from the same rock (*n* = 12) and willow (*n* = 9) ptarmigan individuals that were hosts to lice in our dataset. Laboratory methods and bioinformatic pipelines follow DaCosta and Sorenson (2014; Python scripts available at http://github.com/BU-RAD-seq/ddRAD-seq-Pipeline) and Lavretsky et al., (2015). With one exception, libraries were prepared with dual 6 nt indices and demultiplexed using bcl2fastq v.2.20 or MiSeq Reporter software (Illumina). Single‐end, 150 base‐pair sequencing was completed on either an Illumina HiSeq 4000 or MiSeq. Raw Illumina reads have been accessioned on the National Center for Biotechnology Information (NCBI) Sequence Read Archive (BioProject PRJNA634168; SAMN14995710–SAMN14995731). See Sonsthagen and Wilson (2020) for ddRAD accession information by sample. Final output files consisting of all bi‐allelic SNPs were generated with custom python scripts (Lavretsky et al., 2016). Specifically, to limit any biases due to sequencing error and/or allelic dropout, a minimum of 10 reads was required to score a locus as heterozygous. Chromosomal positions across markers were attained by BLAST to the chicken genome (*Gallus gallus*, assembly version 5.0, GenBank assembly reference GCA_000002315.3). Analyses were restricted to autosomal markers only. This SNP‐calling pipeline generated 4,182 SNPs (0% missing) for willow ptarmigan, and 5,476 SNPs (0% missing) for rock ptarmigan.

### 2.6 Population genetic analysis

We performed several analyses to compare the population structures of willow and rock ptarmigan and their lice. First, we analyzed the SNPs in principal component analyses (PCA) with the default parameters using the “glPca” command from the *adegenet* v.2.1.1 R package (Jombart, 2008). We also assessed population structure for these groups using ADMIXTURE v.1.3.0 (Alexander, Novembre, & Lange, 2009). We ran ADMIXTURE for *K* = 2–6 and tested for optimal values of K using the cross‐validation procedure (cv = 5).

We tested for isolation by distance (IBD) among individuals in the three louse OTUs and in both ptarmigan species. For each group, we converted the SNPs to Euclidean distance matrices and the sampling localities to geographic distance matrices using Geographic Distance Matrix Generator v.1.2.3 (http://biodiversityinformatics.amnh.org/open_source/gdmg). We then compared the genetic and geographic distances using Mantel tests in *adegenet* with 999 permutations.

To statistically compare population genetic structure between ptarmigan and their lice, we used genetic distances based on SNPs and Mantel tests, following Feurty et al. (2016). We again converted SNPs to Euclidean distances for the ptarmigan and lice, and then ran Mantel tests in *adegenet* with 999 permutations. Because there are three rock ptarmigan individuals that are likely related (REW895, REW896, REW897 in Table S1), we ran three additional Mantel tests with only one ptarmigan and louse representative from these three individuals (one test for each representative). To account for any correlations driven by IBD, we also ran partial Mantel tests with 999 permutations on the SNP distance matrices in the R package *vegan* v.2.5.6 (Oksanen et al., 2019), using the geographic distance matrices to control for IBD.

## 3 RESULTS

### 3.1 Phylogenetic relationships among *Lagopoecus* and *Goniodes*

Concatenated, coalescent, and COI phylogenies for *Lagopoecus* and *Goniodes* lice showed largely concordant relationships across analyses. OTU analyses based on the COI and concatenated phylogenies indicated eight OTUs for *Lagopoecus* and five OTUs for *Goniodes*.

**Figure 3:**
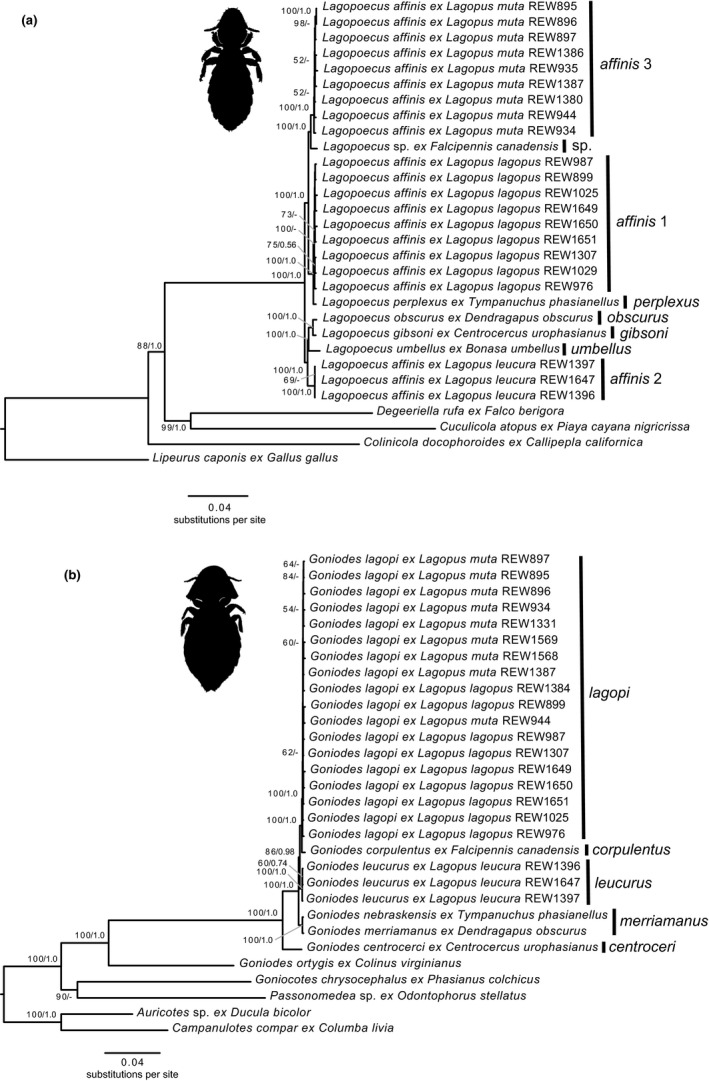
Phylogenetic trees of (a) *Lagopoecus* and (b) *Goniodes* lice from grouse based on concatenated alignments of 619 and 753 nuclear genes, respectively. Bootstrap values (BS) >50% and local posterior probabilities (PP) >0.5 from a coalescent analysis of gene trees are indicated above or below branches of the tree (BS/PP). OTUs are indicated to the right of the tip labels. Individual tips are labeled with the host codes from Table S1

The eight OTUs within *Lagopoecus* were each associated with a single host species: *L. gibsoni* from greater sage‐grouse, *L. obscurus* from dusky grouse, *L. perplexus* from sharp‐tailed grouse, *L. umbellus* from ruffed grouse, and undescribed species (*L*. sp.) from spruce grouse, and three different host‐specific and polyphyletic taxa within *L. affinis* (hereafter *affinis* 1–3) from willow, white‐tailed, and rock ptarmigan, respectively. We recovered *L. obscurus* and *L. gibsoni* as sister species, and these two species + *L. umbellus* as sister to *L. affinis* 3. We recovered this clade as sister to a clade containing *L. affinis* 1 + *L. perplexus* and *L. affinis* 2 + *L*. sp. from spruce grouse. Most of these relationships were recovered with high support (>100% bootstrap, >1.0 local posterior probability) (Figure 3a, Figure S1). Although the COI phylogeny showed an identical topology among OTUs, the bootstrap support was much lower overall (five branches received < 70% bootstrap) (Figure S2).

For *Goniodes*, we recovered separate OTUs for *G. centrocerci* from greater sage‐grouse and *G. corpulentus* from spruce grouse. We also recovered *G. merriamanus* from dusky grouse and *G. nebraskensis* from sharp‐tailed grouse as a single OTU, *G. leucurus* from white‐tailed ptarmigan as a single OTU, and *G. lagopi* from both willow and rock ptarmigan as a single OTU. We recovered *G. lagopi* as sister to *G. corpulentus*, and these two OTUs as sister to *G. leucurus*. We recovered this clade as sister to *G. merriamanus* + *G. nebraskensis*. We recovered *G. centrocerci* as sister to the rest of the *Goniodes* in‐group. As with *Lagopoecus*, most of the relationships among the *Goniodes* OTUs in the concatenated and coalescent phylogenies received > 100% bootstrap and >1.0 local posterior probability support, and the COI phylogeny had an identical topology but with lower overall support (Figure 3b, Figures S3‐S4).

### 3.2 Cophylogenetic analysis

Testing for congruence between the *Lagopoecus* and *Goniodes* OTU phylogenies (each OTU collapsed to a single branch) and the host phylogeny produced mixed results. With distance‐based analyses, PACo indicated overall congruence in both systems (*Goniodes*: *p* = .020, $m_{\text{XY}}^{2}$ = 0.006; *Lagopoecus*: *p* = .005, $m_{\text{XY}}^{2}$ = 0.007) whereas ParaFit did not indicate significant congruence (*Goniodes*: *p* = .09, ParaFitGlobal = 2.28e^−6^; *Lagopoecus*: *p* = .15, ParaFitGlobal = 2.42e^−6^). However, in both *Goniodes* and *Lagopoecus*, none of the individual associations showed significant contributions to the overall congruence based on the corrected ParaFit link tests (Table S2) or median PACo jackknife residuals (Figures S5‐S6).

**Figure 4:**
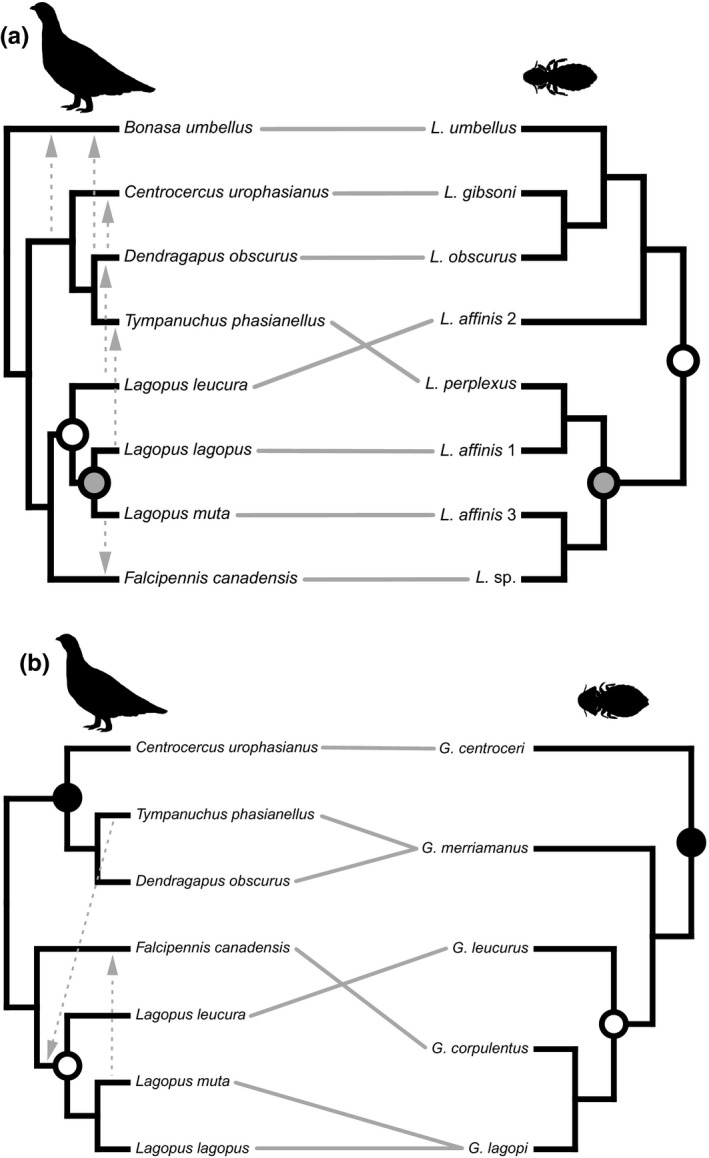
Tanglegram depicting the cophylogenetic relationships between grouse and their parasitic lice in the genera (a) *Lagopoecus* and (b) *Goniodes*. Gray lines connect associated species. Shaded circles indicate cospeciation events recovered from reconciliation analyses, with corresponding grouse and louse divergences filled with the same level of shading. The dotted arrows indicate host switches recovered from the same analyses

The reconciliation analysis for *Lagopoecus* indicated that this louse clade originated in the common ancestor of ptarmigan. The phylogenetic reconciliation recovered two cospeciations (one between the ancestor of ptarmigan and their lice, and a second between the ancestor of willow and rock ptarmigan and their lice), five host switches (from rock ptarmigan to spruce grouse, from willow ptarmigan to sharp‐tailed grouse, from white‐tailed ptarmigan to dusky grouse, from dusky grouse to ruffed grouse, and from dusky grouse to greater sage‐grouse), and one loss (Figure 4a, Table S3). The total observed cost was 10, which was not significantly lower than the randomizations (*p* = .46). Reconciliation analysis for *Goniodes* indicated the louse clade originated in the common of ancestor of *Centrocercus*,* Tympanuchus*, and *Dendragapus*. This analysis recovered two cospeciation events (one between the ancestor of *Centrocercus*,* Tympanuchus*, and *Dendragapus* and their lice, and a second between the ancestor of ptarmigan (*Lagopus*) and their lice), two host switches (one from rock ptarmigan to spruce grouse, and a second from sharp‐tailed grouse to the ancestor of ptarmigan), and two failures to diverge (Figure 4b, Table S3). The total observed cost was 6, which was not significantly lower than the cost of randomized associations (*p* = .07). The unconstrained Jane analysis (without divergence information of the hosts) for *Goniodes* indicated these lice originated in the common of ancestor of *Centrocercus*,* Tympanuchus*, and *Dendragapus*. The analysis recovered two cospeciation events, two host switches, and two failures to diverge (Figure S8, Table S3). The total observed cost was 6, which was not significantly lower than the cost of randomized associations (*p* = .07). The Jane analysis for *Lagopoecus* indicated these lice originated in the common ancestor of *Lagopus*. The phylogenetic reconciliation recovered three cospeciations, four host switches, and one loss (Figure S7, Table S3). The total observed cost was 9, which was not significantly lower than the randomized reconcilliations (*p* = .46).

### 3.3 Population genomic structure

PCA did not show obvious structure for *G. lagopi*, although there was a slight distinction between lice from rock ptarmigan and willow ptarmigan along PC1 (Figure 2). The ADMIXTURE cross‐validation analysis indicated *K* = 6 was the optimal value of *K*, but most of the ancestry at the level of *K* had values ≪ 0.01 (Figure S9). The next optimal value was *K* = 1 (i.e., lack of structure). PCA for *L. affinis* 1 and 2 also did not show substantial structure and had similar results with ADMIXTURE (high optimal values of *K* with negligible mixed ancestry and next highest values of *K* = 1). However, *L. affinis* 3 samples from the Thompson Pass rock ptarmigan population (REW895, REW896, REW897) were distinctive, and one individual (REW897) appeared to have mixed ancestry with this population and the other two populations (Arctic and Denali). ADMIXTURE showed similar patterns at *K* = 6. An individual *G. lagopi* louse from a different Thompson Pass ptarmigan (REW896) showed a similar mixed‐ancestry pattern in the PCA and ADMIXTURE results. For the ptarmigan, PCA and ADMIXTURE showed limited structure except the potential familial group of rock ptarmigan in Thompson Pass (Figure 4, Figure S9).

Tests for IBD were significant for *L. affinis* 3 (*r* = .39, *p* = .005) but removing two of the Thompson Pass individuals resulted in nonsignificant results (*r* = .16, *p* = .147). IBD tests were not significant for *L. affinis* 1 (*r* = −.12, *p* = .674) or *G. lagopi* (*r* = .01, *p* = .466; *r* = −.05, *p* = .676 with two Thompson Pass individuals removed). Tests for *G. lagopi* separated by host species were also not significant (willow ptarmigan lice: *r* = −.04, *p* = .573; rock ptarmigan lice: *r* = .18, *p* = .207; *r* = −.07, *p* = .652 with two Thompson Pass individuals removed). In contrast, IBD tests for both willow ptarmigan (*r* = .50, *p* = .001) and rock ptarmigan (*r* = .44, *p* = .022) were significant. The result remained significant for rock ptarmigan even with two Thompson Pass individuals removed (*r* = .28, *p* = .029).

Mantel tests showed significant correlations between the rock ptarmigan and both the *L. affinis* 3 (*r* = .65, *p* = .013) and *G. lagopi* (*r* = .50, *p* = .005) genetic distances. However, when we removed two of the potentially closely related Thompson Pass birds (removing two of REW895‐897) and their lice, the Mantel tests were no longer significant (*Lagopoecus*: *r* = −.33, −0.29, −0.54; *p* = .800, 0.752, 0.946; *Goniodes*: *r* = −.49, .05, −.59; *p* = .883, .583, .916; numbers for tests run with representative REW895, REW896, and REW897, respectively). Mantel tests between willow ptarmigan and their lice were not significant (*L. affinis* 1: *r* = .06, *p* = .408; *G. lagopi*: *r* = −.37; *p* = .947). Partial Mantel tests accounting for IBD did not show any differences from any of the Mantel tests (*L. affinis* 3: *r* = .58, *p* = .013; *r* = −.40, *p* = .85 with two Thompson Pass individuals removed; *G. lagopi*: *r* = .48, *p* = .006; *r* = −.49, *p* = .882 with two Thompson Pass individuals removed; *L. affinis* 1: *r* = .14, *p* = .338; *G. lagopi*: *r* = −.42, *p* = .984).

## 4 DISCUSSION

### 4.1 Insights into the coevolutionary history of grouse and their lice

The comparative evolutionary histories between Arctic Galliformes and their parasitic lice suggest consistent movement of lice among different host species and populations in Alaska. Both genera (*Lagopoecus* and *Goniodes*) of lice had similar patterns of evolutionary history with their hosts. The lice generally showed incongruent phylogenetic relationships with their hosts, and population structure was not correlated between populations of rock and willow ptarmigan and their lice. This incongruence may reflect ancestral host distributions rather than host diversification. For example, there are two major clades of *Lagopoecus* associated with hosts with primarily northern distributions (rock ptarmigan, willow ptarmigan, spruce grouse, and sharp‐tailed grouse) versus those whose ranges extend into southwestern United States (dusky grouse, greater sage‐grouse, and white‐tailed ptarmigan), including the widely distributed ruffed grouse. The only exception is sharp‐tailed grouse (also a prairie grouse) which is thought to have diverged in the southwestern Nearctic with subsequent dispersal into more northern regions (Drovetski, 2003). Phylogenetic reconciliations of galliform hosts and lice also showed evidence for multiple host switching events in each louse genus, which may reflect more contemporary dispersal patterns. However, there is still evidence for codivergence in both *Lagopoecus* and *Goniodes*. In particular, there is a shared cospeciation event between the common ancestor of ptarmigan and their lice, and a second cospeciation between *Lagopoecus* and the ancestor of willow and rock ptarmigan. If these reconciliations are accurate, they suggest earlier lineages of these lice diverged with their ptarmigan hosts and subsequently switched to other galliform hosts in geographic proximity. Considering these patterns at both macro‐ and microevolutionary scales, it seems the lice are a valuable source of information for understanding galliform evolutionary history and population dynamics.

At the macroevolutionary scale, the relationship of lice and their galliform hosts suggests a history of galliform dispersal and interspecies interactions. This is in agreement with previous studies that indicate grouse and ptarmigan have dispersed over large biogeographic areas (e.g., from North American to Eurasia) throughout their evolutionary history (Drovetski, 2003; Persons et al., 2016). Although grouse and ptarmigan tend to be nonmigratory and are likely separated by microhabitat differences throughout most of the year (Hoffman & Braun, 1975; Rodewald, 2020), many species undergo local seasonal movements related to such factors as food supply and snow conditions (Irving et al., 1967; Schroeder & Braun, 1993; Tape, Lord, Marshall, & Ruess, 2010). These localized patterns of movement may facilitate interspecies interactions through direct or indirect contact. Because we detected many host switches within Alaska, our results suggest mixed‐species interactions likely occur with relative frequency among different species of grouse. Although seeing all ptarmigan or grouse species of Alaska in the same location is not uncommon, grouse and ptarmigan interactions should be less frequent because they have different habitat requirements. However, grouse and ptarmigan species are regularly observed in close proximity to each other (within 5 km) and in some cases the same location at the edges of preferred habitat (e.g., spruce grouse and willow ptarmigan; Denali Highway, Alaska) (Montgomerie & Holder, 2020; Moss, 1972; Sonsthagen & Wilson, 2020). Interactions do not necessitate physical contact between different species for parasite transfer. Although feather lice do not survive for long periods of time away from their hosts (usually no longer than a few days; Marshall, 1981; Rothschild & Clay, 1952), it is possible for lice to switch hosts due to different host species sharing dust baths or nesting sites (Clay, 1949; Timm, 1983). Thus, host switching may only require proximity between different host species with similar habitat preferences.

Comparative population structure (microevolutionary scale) between willow and rock ptarmigan and their lice was particularly informative about potential contact between different populations of these hosts. Both species of ptarmigan suggest some level of population structure and/or isolation by distance, even when accounting for potentially closely related individuals (it should be noted that host population structure should be interpreted with caution due to low sample size). However, no structure was detected within either genus of louse on these hosts. Together, these patterns (host and parasite) suggest there is still contact or at least distributional overlaps in ptarmigan populations which may not be perceptible from the host data alone. This is evident when looking at lice from Thompson Pass (near Valdez, Alaska), which is geographically closer to the Denali population, but *Lagopoecus* lice from Denali cluster with lice from the more distant Arctic population. In addition, a single Thompson Pass individual louse (from REW897) appears to have mixed ancestry in both the Thompson Pass and Denali/Arctic populations, and a *Goniodes* louse from a different Thompson Pass individual (REW896) shows a similar signal. This suggests that there is some amount of dispersal or connectivity linking subarctic and Arctic ptarmigan. It is possible the discordance in structure between ptarmigans and their lice is an effect of “straggling” or incomplete lineage sorting in the lice (Ròzsa, 1993). That is, if ptarmigan populations have recently diverged from each other, then the lice may not yet reflect this structure in their hosts. However, because there were several glacial refugia in Alaska during the Last Glacial Maximum (Carrara, Ager, & Baichtal, 2007; Shafer, Cullingham, Côté, & Coltman, 2010), many ptarmigan populations likely have a relatively ancient divergence. Additionally, given the higher rates of molecular divergence in lice relative to their hosts, as a result of the shorter generation times in lice (Hafner et al., 1994), and the overall lack of population structure in both *Lagopoecus* and *Goniodes*, our results are more likely related to the dispersal of lice among ptarmigan populations.

### 4.2 Factors influencing the relationships between grouse and their lice

In addition to ptarmigan and grouse ecology shaping the evolutionary relationships with their lice, there are other factors that could drive the observed patterns. For example, although feather lice are tightly associated with their hosts, it is possible for lice to disperse independently of their host. For example, 33 species of lice have been documented to use winged hippoboscid flies as a dispersal mechanism, a phenomenon more broadly known as “phoresy” (Bartlow, Villa, Thompson, & Bush, 2016). Experimental, phylogenetic, and population genetic evidence strongly suggests the ability to use hippoboscid flies has a great effect on the evolutionary patterns between birds and lice (Clayton & Johnson, 2003; DiBlasi et al., 2018; Harbison & Clayton, 2011; Harbison, Jacobsen, & Clayton, 2009; Sweet & Johnson, 2018). Phoresy has not been recorded for any of the species of lice in this study, but phoresy has been documented in a species of *Lagopoecus* (*L. lyrurus*) from black grouse (*Tetrao tetrix*) (Forsius, 1912), a species distributed in Eurasia. There are also records of hippoboscid flies in Alaska (specifically *Ornithomyia pallida*) associated with several of the grouse genera included in this study (*Bonasa*,* Dendragapus*, and *Lagopus*) (Maa, 1969). If phoresy does occur in *Lagopoecus* but not *Goniodes*, this could explain the difference in the number of host switches recovered in each genus (two in *Goniodes* versus five in *Lagopoecus*). However, there are other patterns that are contrary to what we would expect given a difference in dispersal ability. *Lagopoecus* is more host‐specific than *Goniodes* and has a higher species‐level diversity even though, based on the phylogenetic reconciliation, the genus originated more recently in this grouse clade. If there are differences in dispersal ability between comparable groups of lice (i.e., from the same groups of hosts), we expect the parasites more capable of dispersal to show less host specificity (DiBlasi et al., 2018; McCoy, Boulinier, & Tirard, 2005; Stefka, Hoeck, Keller, & Smith, 2011; Sweet & Johnson, 2018). Nevertheless, at a population level, both genera of ptarmigan lice show a similar lack of structure, suggesting there are not necessarily differences in dispersal ability, at least not among populations. Overall, further work is needed to determine whether phoresy occurs in *Lagopoecus* and *Goniodes* from North American grouse, as this could help to explain some of the observed evolutionary patterns between these lice and their hosts.

Host body size is another factor that could shape cophylogenetic patterns between hosts and parasites. Previous work in lice and other parasites has shown instances where body size is positively correlated between hosts and their parasites, a pattern known formally as Harrison's Rule (Harrison, 1915; Johnson, Bush, & Clayton, 2005). These limitations imposed by host body size can dictate host switching (Clayton, Bush, Goates, & Johnson, 2003). If a parasite is adapted to live on a host species with a particular body size, it will be very difficult for that parasite to establish populations on a different host species with a much larger or smaller body size. Experimental research has shown this to occur in wing lice from pigeons; lice transferred to different‐sized pigeons were often unable to avoid preening behavior and were quickly removed by the host (Bush & Clayton, 2007; Clayton et al., 2003). This can lead to reproductive isolation between populations of lice and ultimately to speciation (Villa et al., 2019). However, host body size is likely not a crucial factor for grouse and their lice. Although there is some variation in body size among different species of these Galliformes, host switching does not seem to be limited by size differences. In particular, we recovered a host switch from one of the smallest grouse species (white‐tailed ptarmigan, 300–400 g; Martin, Robb, Wilson, & Braun, 2015) to one of the larger species (dusky grouse, >1,000 g; Zwickel & Bendell, 2018). Similarly, our phylogenetic reconciliations between these Galliformes and their lice revealed evidence for several host switches between different host genera, but no host switches within a genus. This pattern likely eliminates topology‐based explanations of host switching, such as clade‐limited host switching (Sorenson, Balakrishnan, & Payne, 2004).

Although *Lagopoecus* and *Goniodes* lice from Alaska Galliformes have similar cophylogenetic and population genetic patterns, there are also considerable differences between the two genera. *Lagopoecus* has more host switches than *Goniodes*, which as discussed above could be attributed to differences in dispersal ability. Both genera have two cospeciation events with their hosts, including a cospeciation within the ptarmigan (*Lagopus*) genus, but neither of these events are shared between the two louse genera. This implies that even though the two genera are associated with same group of hosts, they have fairly separate and independent evolutionary histories. The phylogenetic reconciliations give further support for this independence, as the two genera appear to originate at different times and within different genera of grouse or ptarmigan. *Lagopoecus* was recovered as originating in ptarmigan, with subsequent host switches to other genera of grouse. Conversely, *Goniodes* originated in the grouse (*Centrocercus/Tympanuchus/Dendragapus*) clade, with a subsequent host switch to ptarmigan. Both *Lagopoecus* and *Goniodes* are associated with hosts outside of the scope of this study, so future work based on a broader geographic and taxonomic sampling is needed to more clearly determine the evolutionary history of these louse genera.

### 4.3 The potential perils of uninformed cophylogenetic analyses

The results of our cophylogenetic analyses between Galliformes and their *Lagopoecus* and *Goniodes* lice show compelling patterns related to host–parasite coevolution. These patterns largely make sense in light of the biology of both the hosts and parasites. However, our analyses also revealed potential pitfalls in conducting cophylogenetic analysis without a priori information about relative timing of diversification. Our initial phylogenetic reconciliation analyses produced results for the *Lagopoecus* and *Goniodes* systems that were incompatible with one another. Specifically, it is not possible that *Goniodes* lice switched from the ancestor of grouse (*Dendragapus* and *Tympanachus*) to the ancestor of ptarmigan (*Lagopus*) and that *Lagopoecus* lice switched from white‐tailed ptarmigan to the ancestor of grouse (*Centrocercus*,* Tympanuchus*, and *Dendragapus*). The split between grouse (*Centrocercus*, *Tympanachus*, and *Dendragapus*) cannot have occurred both before and after the basal split in crown ptarmigan (*Lagopus*) (Figures S7–S8). One, or both, of the reconciliations has to be incorrect. It is possible these inconsistencies are a result of stochasticity in small sample sizes (i.e., relatively few host–parasite associations). However, there were no incompatible host switches once we constrained possible host switches based on divergence time estimates of the hosts. Using divergence times in cophylogenetic analyses has been advocated by several studies and reviews of the field, particularly for confirming the legitimacy of cospeciation (Cruaud & Rasplus, 2016; Martínez‐Aquino, 2016; de Vienne et al., 2013). Here, we further demonstrate that it is incredibly useful for reconstructing host switches as well. We recommend that all phylogenetic reconciliations use host and/or parasite divergence time estimates to inform analyses when possible. Our findings also provide an additional reason for comparing cophylogenetic patterns in multiple groups of parasites from the same group of hosts: the different parasite groups can help to corroborate the validity of reconciliation analyses. We suggest all future research pursing cophylogenetic questions in multiparasite systems should carefully evaluate their results for inconsistent evolutionary scenarios among different groups of parasites.

## 5 CONCLUSIONS

Using several species of grouse and two genera of their lice, we have contributed evidence that the evolution and ecology of both the hosts and their parasites have influenced the coevolutionary history within this system. Discord in host–parasite phylogenetic and population genomic patterns in both generalist (*Goniodes*) and more host‐specific (*Lagopoecus*) lice indicate that host movement and interspecies interactions play a strong role in shaping louse diversification. Although geographic structure within grouse and louse species was limited, we did detect lice with admixed ancestry, indicative of long‐distance dispersal and more generally illustrating the utility of examining variation in obligate parasites to identify interactions among host populations and species. In light of current environmental changes and potential introduction of novel parasites/pathogens to the Arctic ecosystem, information regarding these relationships is crucial for understanding linkages between populations, host–parasite interactions, and transmission dynamics across the landscape.

## CONFLICT OF INTEREST

None declared.

## AUTHOR CONTRIBUTIONS

**Andrew D. Sweet:** Conceptualization (equal); data curation (equal); formal analysis (equal); investigation (equal); methodology (equal); writing‐original draft (lead); writing–review and editing (equal). **Robert E. Wilson:** Conceptualization (equal); data curation (equal); formal analysis (equal); funding acquisition (equal); investigation (equal); methodology (equal); project administration (equal); writing–original draft (supporting); writing–review and editing (equal). **Sarah A. Sonsthagen:** Conceptualization (equal); data curation (equal); formal analysis (equal); funding acquisition (equal); investigation (equal); methodology (equal); project administration (equal); writing–original draft (supporting); writing–review and editing (equal). **Kevin P. Johnson:** Conceptualization (equal); formal analysis (supporting); funding acquisition (equal); investigation (equal); methodology (equal); writing–original draft (supporting); writing–review and editing (equal).

## Supporting information

Table S1

Supplementary Material

## Data Availability

Raw Illumina sequence files are available from the NCBI SRA database (BioProject PRJNA634168, SAMN14995710–SAMN14995731; BioProject PRJNA635170; SAMN15029953–SAMN15029999), mtDNA sequence data are available from NCBI GenBank (accession numbers MT517178–MT517227), and detailed sample information are provided in Sonsthagen and Wilson (2020). Relevant data files, including voucher photographs, SNP files, multiple sequence alignments, and phylogenetic tree files are available from the Dryad Digital Data Repository (https://doi.org/10.5061/dryad.qfttdz0ds) (Sweet et al., 2020).
