# Supplementary material for: Lousy grouse: Comparing evolutionary patterns in Alaska galliform lice to understand host evolution and host–parasite interactions
Source: Ecol Evol. 2020 Jul 18;10(15):8379–93. doi: 10.1002/ece3.6545 (PMC7417246; doi:10.1002/ece3.6545)
Supplement: Supplementary file 2 — Supplementary Material [file ECE3-10-8379-s002.docx]

**Supplementary Tables and Figures for:**

Lousy grouse: Comparing evolutionary patterns in Alaskan galliform lice to understand host evolution and host-parasite interactions

Andrew D. Sweet, Robert E. Wilson, Sarah A. Sonsthagen, and Kevin P. Johnson

**TABLES**

Supplementary Table S1 (Excel). Specimen information, extraction and library preparation details, Illumina sequencing statistics, locus assembly, and raw sequence data deposition for lice from ptarmigans and grouse. The individuals marked with an asterisk were used as references for read mapping.

Supplementary Table S2. Results from ParaFit link tests between ptarmigan and grouse species and two genera of their parasitic lice (*Goniodes* and *Lagopoecus*). Results are shown from both the ParaFitLink1 (PF1) and ParaFitLink2 (PF2) tests.

| Host | Parasite | PF1 | p-value | PF2 | p-value |
| --- | --- | --- | --- | --- | --- |
| *Lagopoecus* |  |  |  |  |  |
| *Dendragapus obscurus* | *Lagopoecus obscurus* | 2.09E-07 | 0.4363 | 1.86E-03 | 0.4330 |
| *Tympanuchus phasianellus* | *Lagopoecus* *perplexus* | 1.07E-08 | 0.7234 | 9.53E-05 | 0.7233 |
| *Centrocercus urophasianus* | *Lagopoecus gibsoni* | 2.45E-07 | 0.3348 | 2.19E-03 | 0.3334 |
| *Lagopus leucura* | *Lagopoecus* *affinis* 2 | 2.83E-09 | 0.8551 | 2.52E-05 | 0.8549 |
| *Lagopus lagopus* | *Lagopoecus* *affinis* 1 | 3.13E-07 | 0.2641 | 2.80E-03 | 0.2615 |
| *Lagopus muta* | *Lagopoecus* *affinis* 3 | 3.07E-07 | 0.2360 | 2.74E-03 | 0.2349 |
| *Falcipennis canadensis* | *Lagopoecus* sp. | 3.30E-07 | 0.2871 | 2.94E-03 | 0.2851 |
| *Bonasa umbellus* | *Lagopoecus* *umbellus* | 1.37E-06 | 0.0481 | 1.22E-02 | 0.0468 |
|  |  |  |  |  |  |
| *Goniodes* |  |  |  |  |  |
| *Dendragapus obscurus* | *Goniodes merriamanus* | 7.68E-10 | 0.6627 | 2.02E-05 | 0.6627 |
| *Tympanuchus phasianellus* | *Goniodes merriamanus* | 1.53E-10 | 0.6719 | 4.03E-06 | 0.6719 |
| *Centrocercus urophasianus* | *Goniodes centroceri* | 1.82E-06 | 0.0648 | 4.78E-02 | 0.0660 |
| *Lagopus leucura* | *Goniodes* *leucurus* | 1.63E-07 | 0.3880 | 4.28E-03 | 0.3822 |
| *Lagopus lagopus* | *Goniodes* *lagopi* | 3.68E-07 | 0.1292 | 9.68E-03 | 0.1259 |
| *Lagopus muta* | *Goniodes* *lagopi* | 3.39E-07 | 0.1624 | 8.90E-03 | 0.1581 |
| *Falcipennis canadensis* | *Goniodes* *corpulentus* | 1.76E-07 | 0.4847 | 4.62E-03 | 0.4780 |

Supplementary Table S3. Lowest-cost results from a Jane phylogenetic reconciliation analysis between ptarmigan and grouse species and two genera of their parasitic lice (*Lagopoecus* and *Goniodes*). Constrained analyses included information about grouse divergence times, whereas the unconstrained analyses did not.

|  | Cospeciations | Duplications | Host Switches | Losses | Failures to Diverge | Cost |
| --- | --- | --- | --- | --- | --- | --- |
| Constrained |  |  |  |  |  |  |
|  | *Lagopoecus* |  |  |  |  |  |
|  | 2 | 0 | 5 | 0 | 0 | 10 |
|  | *Goniodes* |  |  |  |  |  |
|  | 2 | 0 | 2 | 0 | 2 | 6 |
|  |  |  |  |  |  |  |
| Unconstrained |  |  |  |  |  |  |
|  | *Lagopoecus* |  |  |  |  |  |
|  | 3 | 0 | 4 | 1 | 0 | 9 |
|  | *Goniodes* |  |  |  |  |  |
|  | 2 | 0 | 2 | 0 | 2 | 6 |

**FIGURES**


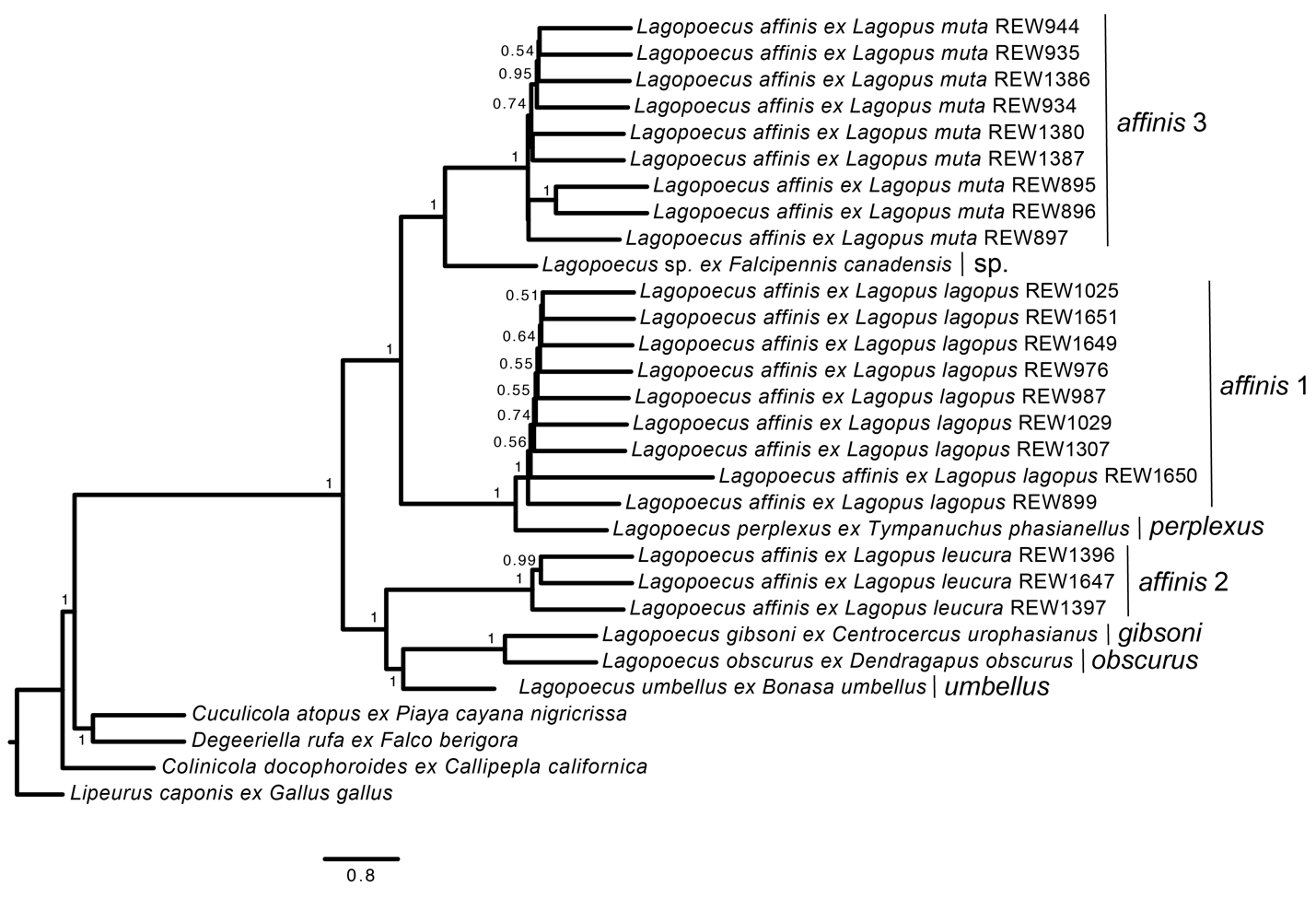


Supplementary Figure S1. Phylogenetic tree from an ASTRAL coalescent analysis based on 617 gene trees of *Lagopoecus* lice from ptarmigan and grouse. Local posterior probability values >0.5 are indicated above the branches. OTUs are indicated to the right of the tip labels. Tips are labeled with the host codes from Supplementary Table S1. Internal branch lengths are in coalescent units, whereas terminal branch lengths are meaningless.


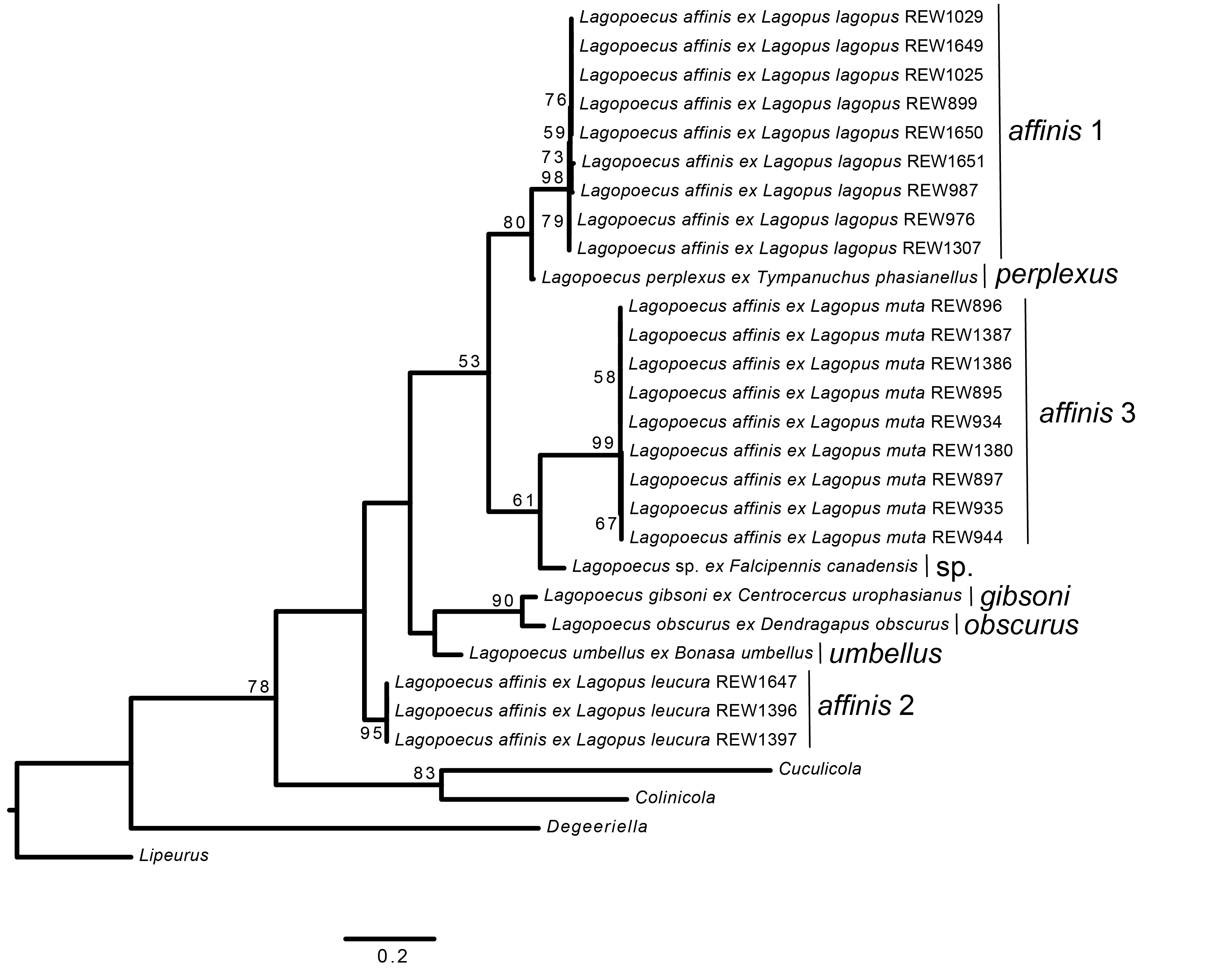


Supplementary Figure S2. Phylogenetic tree based on COI sequences of *Lagopoecus* lice from ptarmigan and grouse. Bootstrap support values >50% are indicated above the branches. OTUs are indicated to the right of the tip labels. Tips are labeled with the host codes from Supplementary Table S1. Branch lengths are in nucleotide substitutions per site.


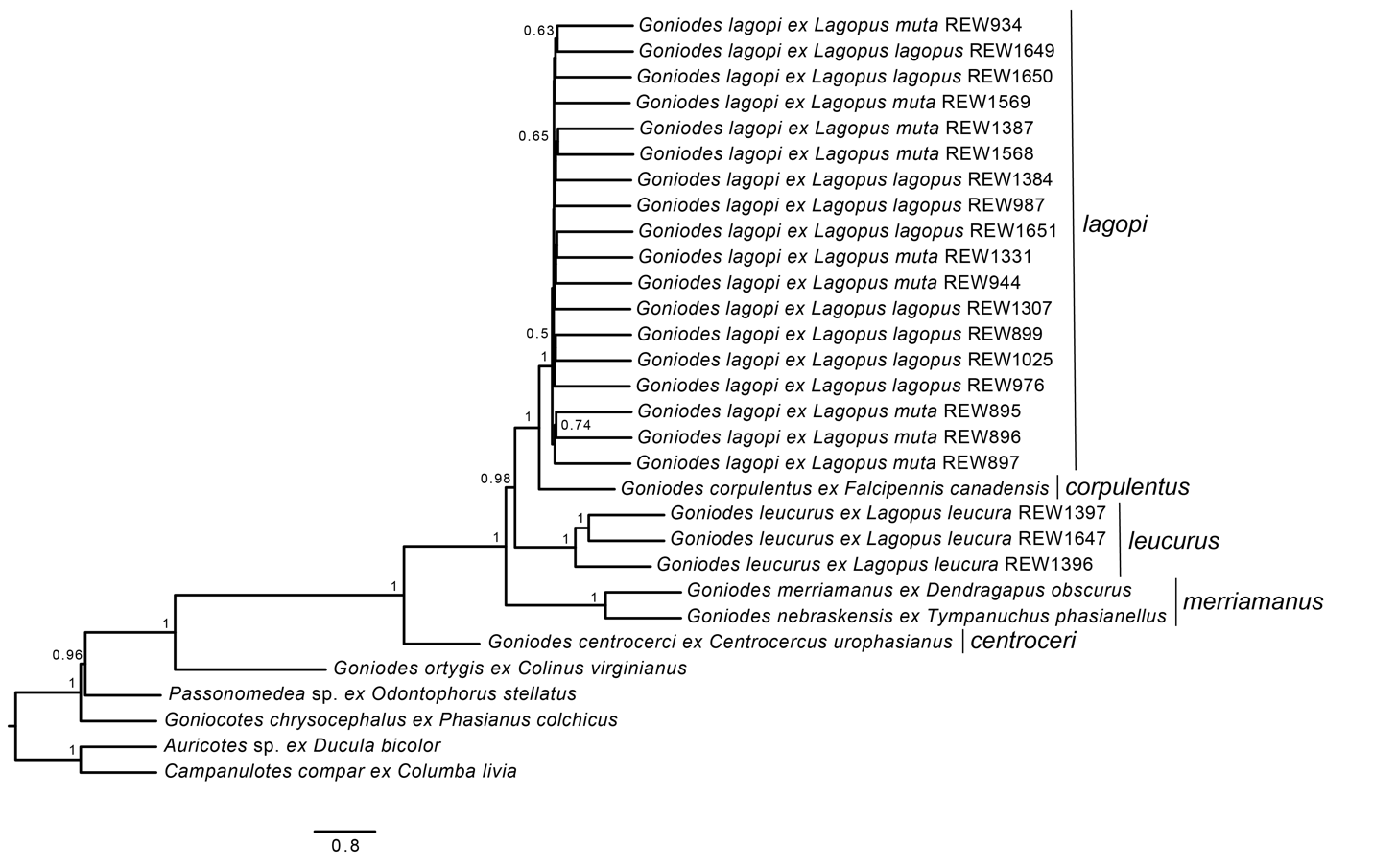


Supplementary Figure S3. Phylogenetic tree from an ASTRAL coalescent analysis based on 753 gene trees of *Goniodes* lice from ptarmigan and grouse. Local posterior probability values >0.5 are indicated above the branches. OTUs are indicated to the right of the tip labels. Tips are labeled with the host codes from Supplementary Table S1. Internal branch lengths are in coalescent units, whereas terminal branch lengths are meaningless.


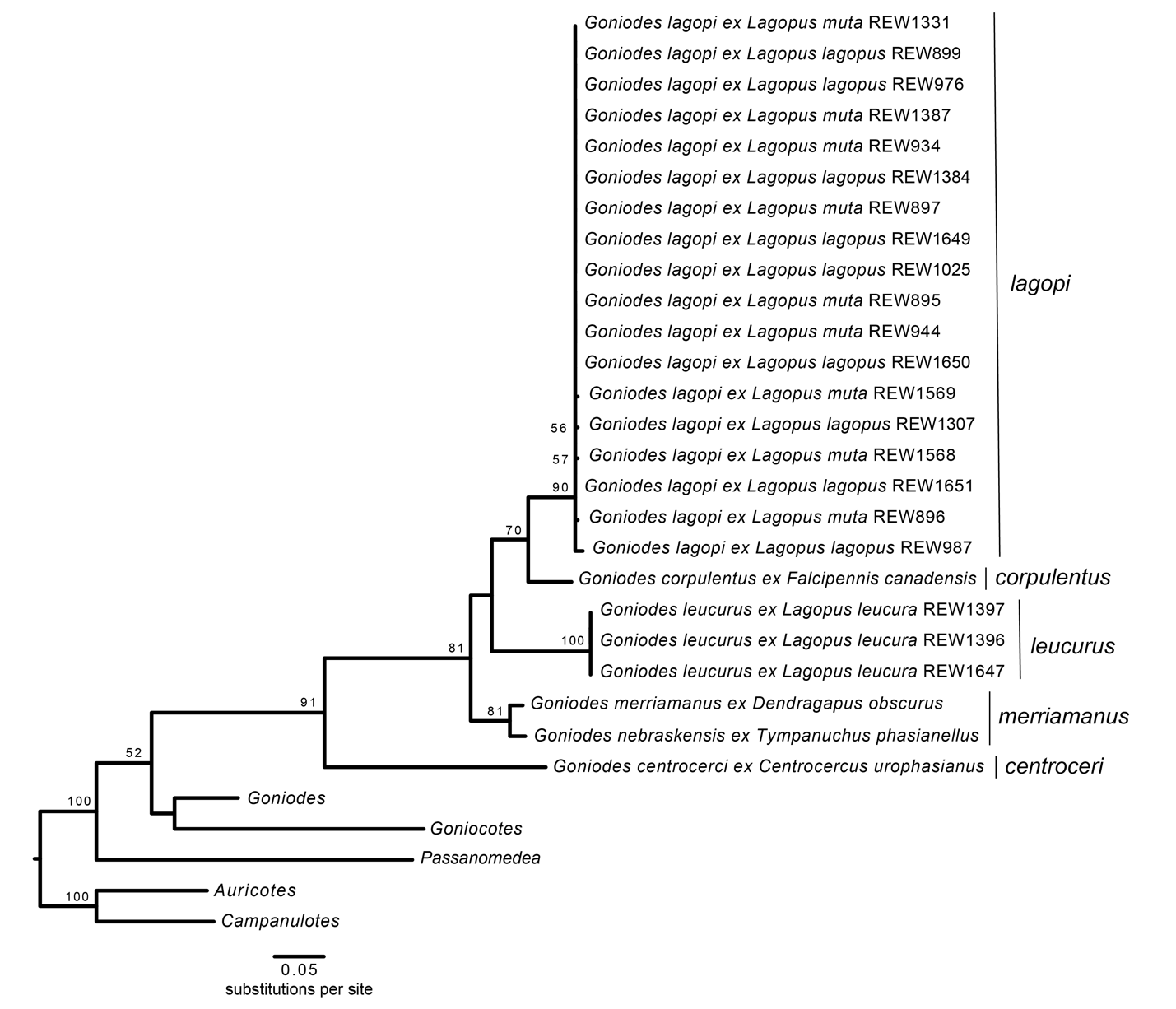


Supplementary Figure S4. Phylogenetic tree based on COI sequences of *Goniodes* lice from ptarmigan and grouse. Bootstrap support values >50% are indicated above the branches. OTUs are indicated to the right of the tip labels. Tips are labeled with the host codes from Supplementary Table S1. Branch lengths are in nucleotide substitutions per site.


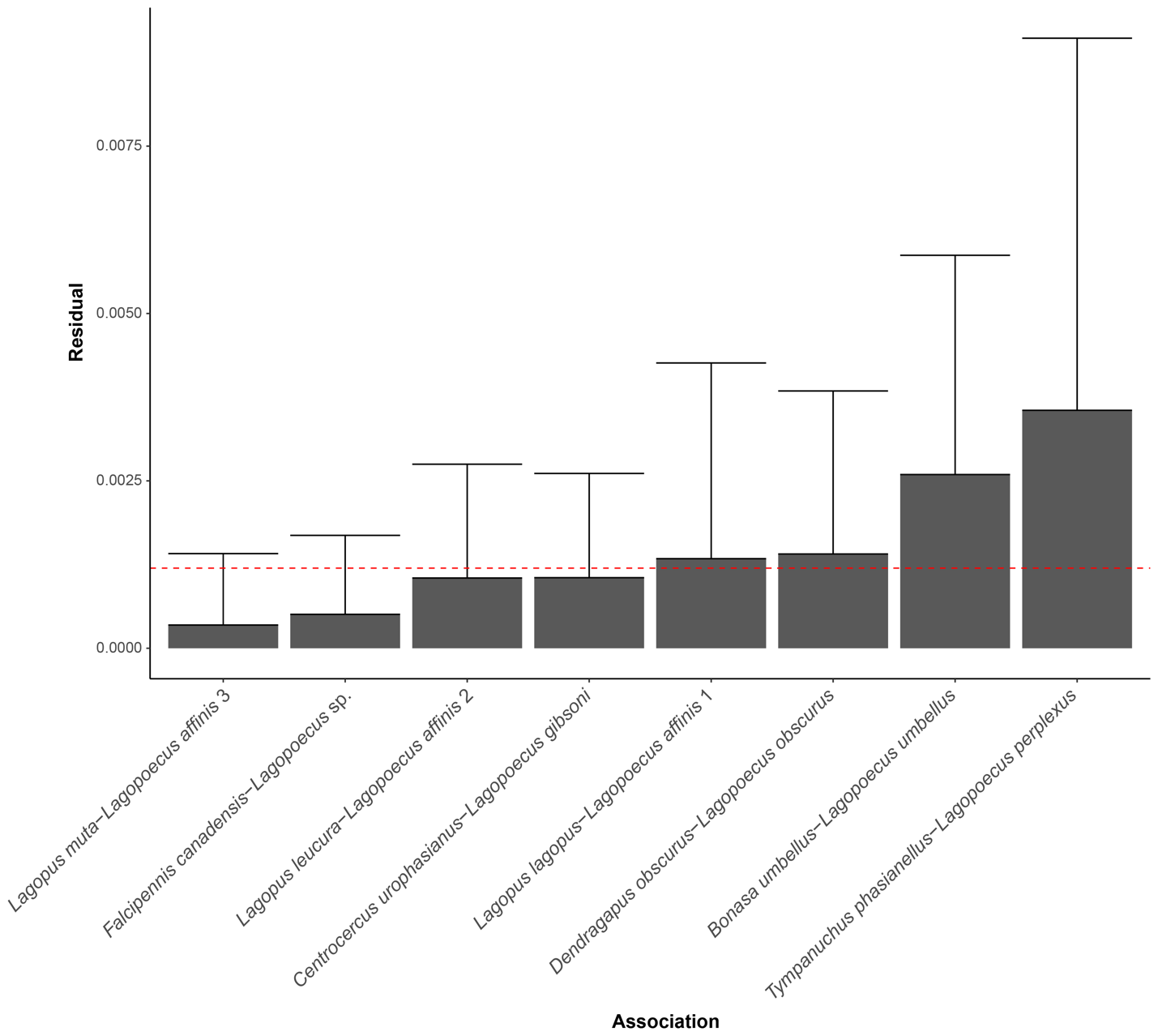


Supplementary Figure S5. Residual values from 9,999 replicates of a PACo cophylogenetic analysis between *Lagopoecus* lice and ptarmigan and grouse. Individual species associations are indicated on the x-axis. The red dotted line indicates the median residual value. Error bars are the 95% confidence intervals.


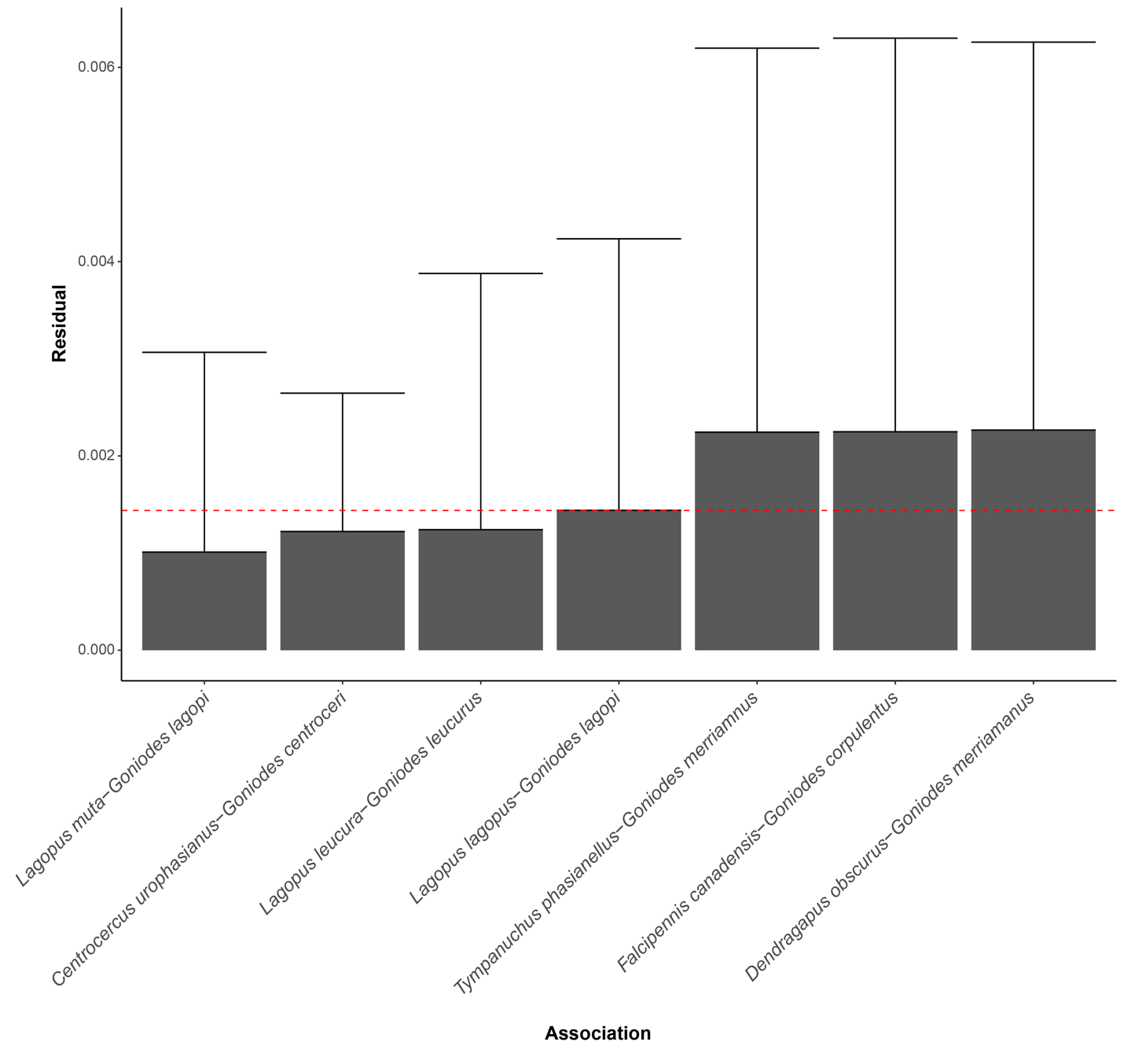


Supplementary Figure S6. Residual values from 9,999 replicates of a PACo cophylogenetic analysis between *Goniodes* lice and ptarmigan and grouse. Individual species associations are indicated on the x-axis. The red dotted line indicates the median residual value. Error bars are the 95% confidence intervals.


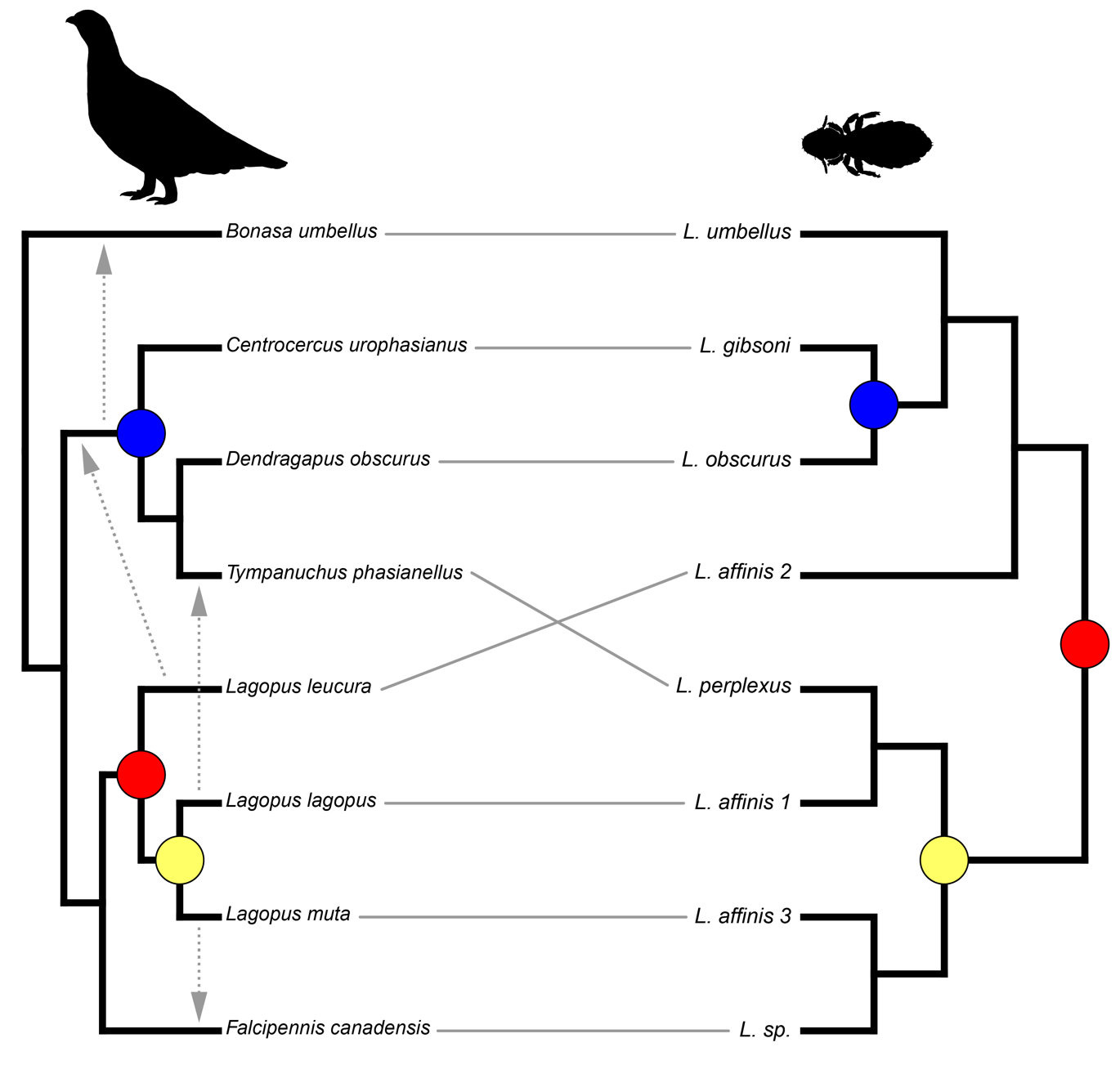


Supplementary Figure S7. Tanglegram depicting the cophylogenetic relationships between ptarmigan and grouse and their parasitic lice in the genus *Lagopoecus*. Gray lines connect associated species, and the thickness of the lines is inversely proportional to the residual values from a PACo cophylogenetic analysis (thicker lines indicates a greater contribution to the overall phylogenetic congruence). Colored circles indicate cospeciation events recovered from an unconstrained reconciliation analysis in Jane (i.e., no host divergence time information), with corresponding grouse and louse divergences filled with the same colors. The dotted arrows indicate host switches recovered from the same analysis.


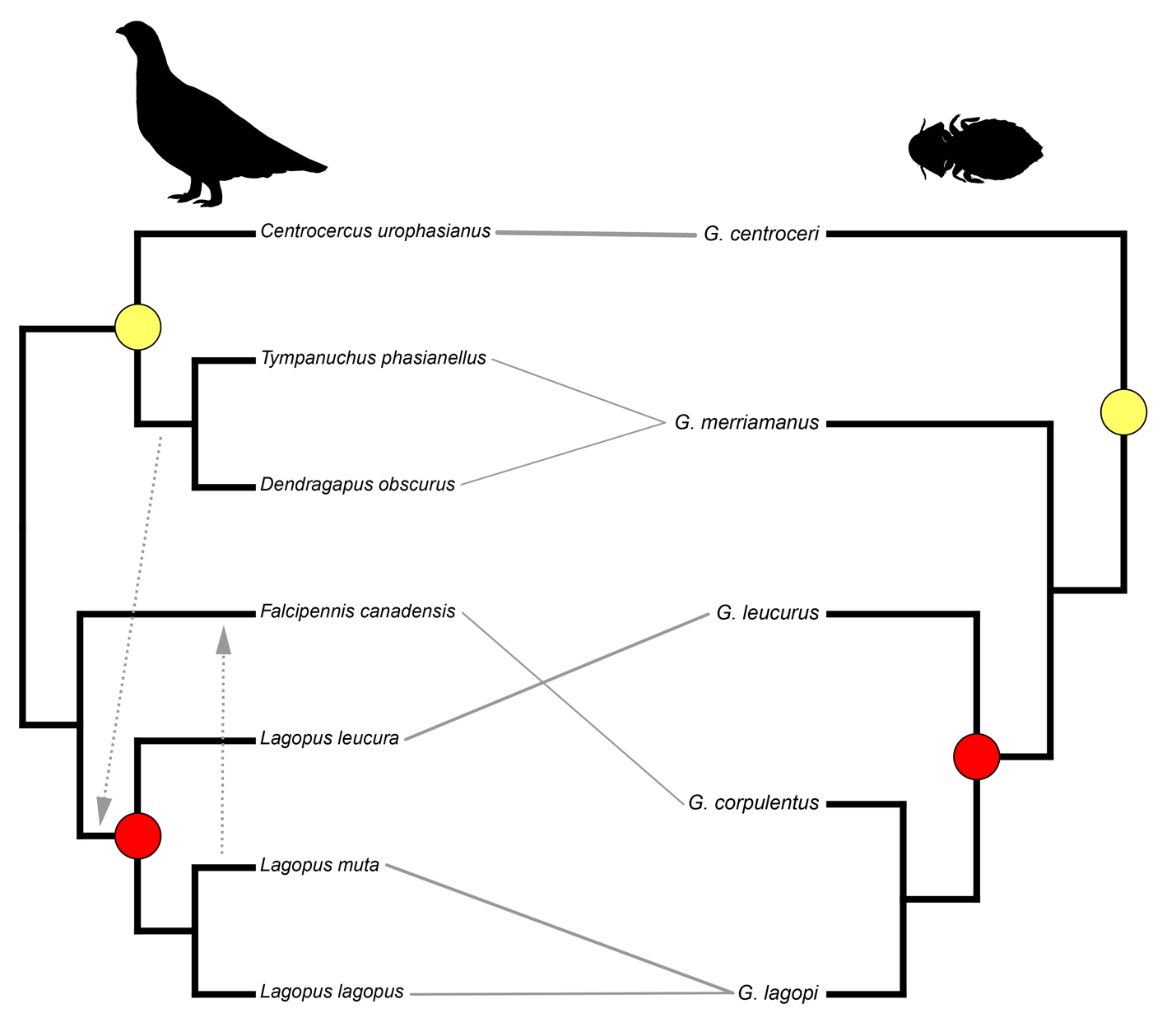


Supplementary Figure S8. Tanglegram depicting the cophylogenetic relationships between ptarmigan and grouse and their parasitic lice in the genus *Goniodes*. Gray lines connect associated species, and the thickness of the lines is inversely proportional to the residual values from a PACo cophylogenetic analysis (thicker lines indicates a greater contribution to the overall phylogenetic congruence). Colored circles indicate cospeciation events recovered from an unconstrained reconciliation analysis in Jane (i.e., no host divergence time information), with corresponding grouse and louse divergences filled with the same colors. The dotted arrows indicate host switches recovered from the same analysis.


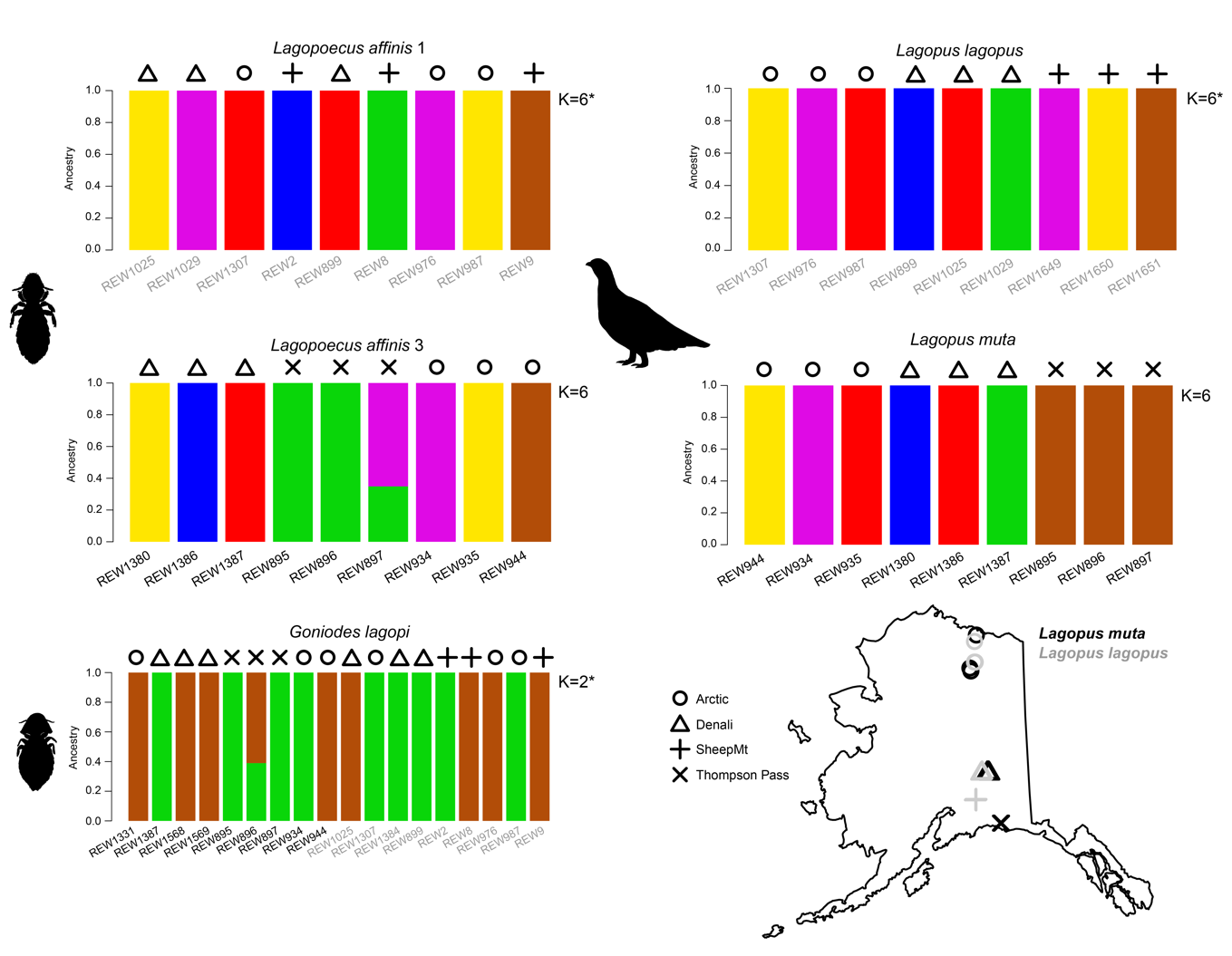


Supplementary Figure S9. ADMIXTURE plots of two species of ptarmigan (*Lagopus muta* and *L. lagopus*) and their lice from the genera *Lagopoecus* and *Goniodes*. Each column represents an individual louse or ptarmigan. The y-axes indicate likelihood of ancestry into a cluster inferred from SNP loci generated from whole or reduced presentation genomic data. Each unique cluster is denoted by a unique color. The number of clusters depicted by each plot are indicated (K); values with asterisks denote plots for which the most likely number of clusters was one. Host codes are indicated below each column in accordance with Supplementary Table S1 and are colored according to ptarmigan species. All individuals were sampled from populations in Alaska, USA; the populations are indicated on the map of Alaska and also above each ADMIXTURE plot column.
